# Supplementary material for: The causal role of circulating inflammatory markers in osteoporosis: a bidirectional Mendelian randomized study
Source: Front Immunol. 2024 Jul 18;15:1412298. doi: 10.3389/fimmu.2024.1412298 (PMC11291241; doi:10.3389/fimmu.2024.1412298)
Supplement: Supplementary file 1 [file DataSheet_1.zip › supplementary material/CIM-OSTEOPOROSIS /GCST90274813.csv_forest.pdf]

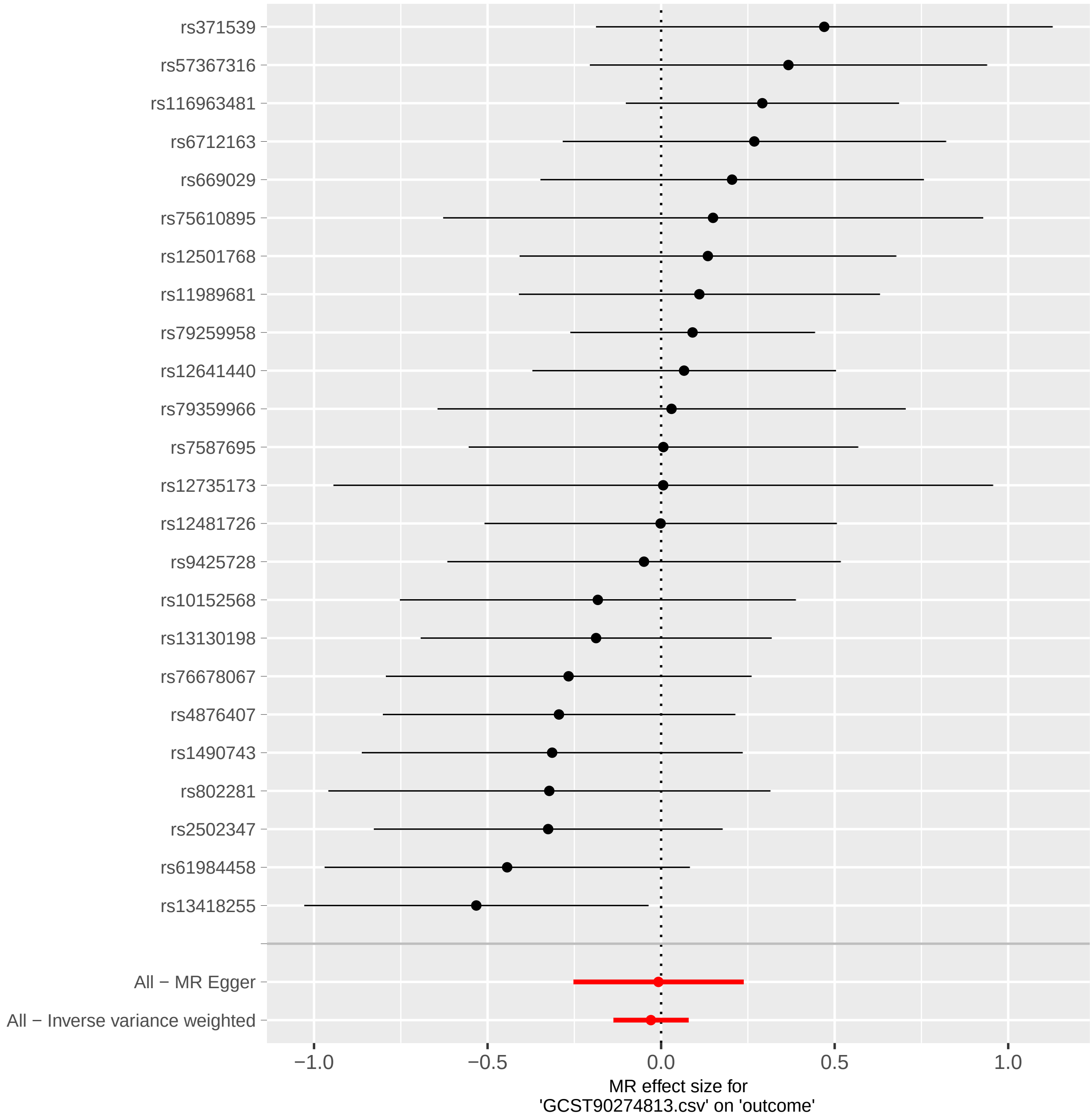
rs371539
rs57367316
rs116963481
rs6712163
rs669029
rs75610895
rs12501768
rs11989681
rs79259958
rs12641440
rs79359966
rs7587695
rs12735173
rs12481726
rs9425728
rs10152568
rs13130198
rs76678067
rs4876407
rs1490743
rs802281
rs2502347
rs61984458
rs13418255
All − MR Egger
All − Inverse variance weighted
−1.0
−0.5
0.0
0.5
1.0
MR effect size for
'GCST90274813.csv' on 'outcome'
